# Neurodynamic evaluation of hearing aid features using EEG correlates of listening effort

**DOI:** 10.1007/s11571-017-9425-5

**Published:** 2017-02-16

**Authors:** Corinna Bernarding, Daniel J. Strauss, Ronny Hannemann, Harald Seidler, Farah I. Corona-Strauss

**Affiliations:** 1 Systems Neuroscience and Neurotechnology Unit, Neurocenter, Saarland University, Medical Faculty & Saarland University of Applied Sciences, School of Engineering, Building 90.5, 66421 Homburg/Saar, Germany; 2 Leibniz–Institute for New Materials, Saarbrücken, Germany; 3 Sivantos GmbH, Erlangen, Germany; 4 MediClin Bosenberg Kliniken, St. Wendel, Germany; 5 Key Numerics GmbH, Saarbrücken, Germany

**Keywords:** Listening effort, Hearing loss, Hearing aids, EEG

## Abstract

In this study, we propose a novel estimate of listening effort using electroencephalographic data. This method is a translation of our past findings, gained from the evoked electroencephalographic activity, to the oscillatory EEG activity. To test this technique, electroencephalographic data from experienced hearing aid users with moderate hearing loss were recorded, wearing hearing aids. The investigated hearing aid settings were: a directional microphone combined with a noise reduction algorithm in a medium and a strong setting, the noise reduction setting turned off, and a setting using omnidirectional microphones without any noise reduction. The results suggest that the electroencephalographic estimate of listening effort seems to be a useful tool to map the exerted effort of the participants. In addition, the results indicate that a directional processing mode can reduce the listening effort in multitalker listening situations.

## Introduction

“Listening effort” can be described as the exertion listeners experience by processing naturally occurring auditory signals in demanding environments (Pichora-Fuller and Singh 2006; McGarrigle et al. 2014). This definition can be complemented by looking closely at the first part of the term ”listening effort”. Kiessling et al. (2003) characterized ”listening” as the process of hearing with intention and attention. Compared to the pure physiological, passive process of hearing which enables access to the auditory system, listening requires mental effort and the allocation of attentional as well as cognitive resources (Hicks and Tharpe 2002; Kiessling et al. 2003; Hornsby 2013). Moreover, this goal-directed attentional effort can be considered as a means to support the optimization of cognitive processes (Sarter et al. 2006).

In case of a hearing loss, the incoming auditory information is degraded by elevated hearing thresholds and a reduced spectrotemporal resolution (Pichora-Fuller and Singh 2006; Shinn-Cunningham and Best 2008). As a result, people with hearing loss have an increased processing effort (Downs 1982; Arlinger 2003). Until now, mainly subjective procedures, like questionnaires (Gatehouse and Noble 2004; Ahlstrom et al. 2013), rating scales (Humes 1999) or self-reports, are applied to estimate listening effort in hearing aid (HA) fitting procedures or in studies related to the assessment of listening effort. Subjective procedures give some indication of the individuals’ perceived listening effort, but it is still uncertain to which extent the subjective data reflect the real experienced effort (Zekveld et al. 2010).

An alternative approach to estimate listening effort objectively are dual task paradigms (Downs 1982; Sarampalis et al. 2009), which are based on a limited capacity model of cognitive resources (Kahneman 1973). The participants have to perform two competing tasks: a primary listening task and a secondary task which is mostly visual or memory related. It is assumed that there is a competition for single limited resources, so that the performance of the secondary task decreases when more resources are allocated in the primary task. This reduction in secondary task efficiency serves as a measure of listening effort. However, this complex method is influenced by many factors such as motivation or task strategy (Hornsby 2013), and requires a considerable cooperation from the participant. Further indications of listening effort, for example the pupil response (Zekveld et al. 2010; Goldwater 1972) and the galvanic skin response (Mackersie and Cones 2011) have been investigated.

Modern HA have settings like noise reduction schemes, which are assumed to ease the speech understanding in complex environments. As a result, the listening effort should be reduced (Lunner et al. 2009). There are a number of studies examining the effects of HA use on listening effort (Downs 1982; Sarampalis et al. 2009; Hornsby 2013; Gatehouse and Gordon 1990; Ahlstrom et al. 2013). The general finding of these studies was that due to the amplification of the relevant auditory information, the audibility of the speech signal was improved resulting in a decreased listening effort.

In previous studies (Strauss et al. 2010; Bernarding et al. 2013), we proposed a new method for the quantification of listening effort by means of evoked electroencephalographic (EEG) activity, which is based on a neurodynamical model. Besides other promising models that can be applied (e.g., Wang et al. 2017), we have used a neurophysical multiscale model which maps auditory late responses as large-scale listening effort correlates. There, we have shown that the instantaneous phase of the N1 component could serve as an index of the amount of listening effort needed to detect an auditory event, such as a target syllable or a toneburst. A higher phase synchronization occurred due to an increased attentional modulation in the range of the theta band, which reflected a higher cognitive effort to solve the auditory task. For more information about the theory of theta-regulated attention, we refer to Haab et al. (2011). In these studies, the N1 component was taken into accout as this component reflects selective attention effects related to an endogenous modulation of the incoming information (Hillyard et al. 1973; Rao et al. 2010; Hillyard et al. 1998). Furthermore, the instantaneous phase of single-trials in the alpha/ theta range was analyzed as it provides more information on the auditory information processing as averaged responses (Brockhaus-Dumke et al. 2008; Ponjavic-Conte et al. 2012). Related to the findings in these studies, it can be assumed that a measure based on the cortical response is an appropriate way to estimate the listening effort. However, there are some limitations in the study of auditory evoked responses (AERs) regarding the design of stimulation paradigms, like the limitation of the auditory stimulation to signals of short duration (Hall 2007, pp. 490ff.) or the dependency on physical stimulus properties (exogenous effects). Therefore, the AERs cannot be analyzed during longer listening periods—for instance during a speech intelligibility test. Furthermore, the exogenous effects have to be minimized. This minimization causes a constraint on the comparability of the results that are to be obtained. This means that the different noise types, SNRs or HA settings, which always modify the incoming auditory signal, cannot be compared directly to each other. To overcome the limitation to signals of short duration, the current study deals with the ongoing oscillatory activity. Here, the EEG can be analyzed during longer listening periods. Thus, the listening effort could be extracted by using noise embedded sentences or during a sentence recognition test. As the HA always alters the auditory signals, different HA features were tested to have varying hearing impressions. Evaluating the estimated effort by a subjective rating scale, we expected to see the same pattern in the subjective and the electroencephalographic estimate. If this would be true, then the influence of the exogenous effects would be minor. These degrees of freedom in the design of the auditory stimulation are essential requirements for a possible prospective EEG-aided HA adjustment in clinical settings.

The link between the previous studies investigating the instantaneous phase of the N1 component and the current study using the instantaneous phase extracted from the ongoing EEG can be achieved via the phase reset model (Sauseng et al. 2007). The phase reset model suggests that the evoked potentials are generated by a phase reset of the ongoing EEG activity. A widely debated topic in the EEG (Kerlin et al. 2010; Ng et al. 2012), electrocorticographic (ECoG) (Zion Golumbic et al. 2013; Mesgarani and Chang 2012) and magnetoencephalographic (MEG) (Peelle et al. 2013; Ding and Simon 2012) research is the phase entrainment of cortical oscillations. Two main hypotheses regarding the functional role of cortical entrainment are under discussion: (1) The cortical entrainment emerges due to physical characteristics of the external stimuli; (2) the phase locking is a modulatory effect on the cortical response triggered by top-down cognitive functions (Ding and Simon 2014). The first theory is supported due to the theta oscillations in the auditory cortex that entrain to the envelope of sound (Ng et al. 2012; Kerlin et al. 2010; Weisz and Obleser 2014). This low-frequency activity can be seen as a reflection of the fluctuations of the speech envelope (Zion Golumbic et al. 2013). The second aspect deals with a modulatory effect on the phase via top-down processes. Here, the synchronization of the phase in auditory processing regions acts like a mechanism of attentional selection (Peelle et al. 2013). This theory of an attentional modulation of the neural oscillations at lower frequencies (4–8 Hz) is supported by studies in the auditory (Kerlin et al. 2010) as well as in the visual domain (Busch and VanRullen 2010). Regarding such a possible attentional effortful modulation of the neural responses via phase locking or synchronization, the proposed method for the extraction of listening effort correlates relies on the instantaneous phase information of the ongoing EEG activity. The hypothesis is that for a non effortful listening environment the phase is rather uniformly distributed on the unit circle than for a demanding condition. For the latter, it is assumed that the phase is more clustered on the unit circle due to an endogenous effortful modulation caused by an increased auditory attention to the relevant auditory signal.

In this work, the proposed EEG method for the extraction of listening effort correlates in people with moderate hearing loss was tested. This was done to examine if the proposed EEG method could serve as a novel measure of listening effort. The new method was evaluated by the results of the subjective listening effort and speech intelligibility scales. Additionally, we investigated the effects of different HA settings on the listening effort. These settings included a new feature which combines a directional microphone technique with a noise reduction algorithm and was tested in a medium and a strong setting. In a further setting, this feature was turned off and a configuration using omnidirectional microphones without any noise reduction was tested.

## Methods

### Ethics statement and recruitment of the participants

The study was approved as scientific study by the local ethics committee (Ärztekammer des Saarlandes; Medical Council of the Saarland). The decisions of the ethics committee are made in accordance with the Declaration of Helsinki.

The participants were recruited from a hearing rehabilitation center. They were informed about the content of the study in a one-to-one appointment. There, the procedures were explained aurally and all questions of the participants related to the procedure and the consent form were answered in detail. After this, all participants provided written informed consent for the investigation and the subsequent data analysis. The participants were compensated for their time by a voucher.

### Participants and inclusion criteria

Two listening conditions were tested in a single session (condition I and II). A total of 14 experienced HA users with a moderate hearing loss participated in this study. All participants reported to wear their own HA regularly in different acoustic environments. We expected that experienced HA users are able to recognize even minor differences between the different HA settings. Furthermore, Ng et al. (2014) showed that new hearing aid users need a higher cognitive processing to understand speech processed by the HA. All 14 participants were native German speakers and attended in condition I of this study (mean age: M $= 65.64$ years (SD $=7.93$ years), seven female/seven male). Two participants quit the experiment after completing condition I. Thus, a total of 12 participants (mean age: M $=66.25$ years (SD $=7.74$ years), five female/seven male) took part in condition II. The participants were included if they had at least 80% artifact free EEG data.

**Fig. 1:**
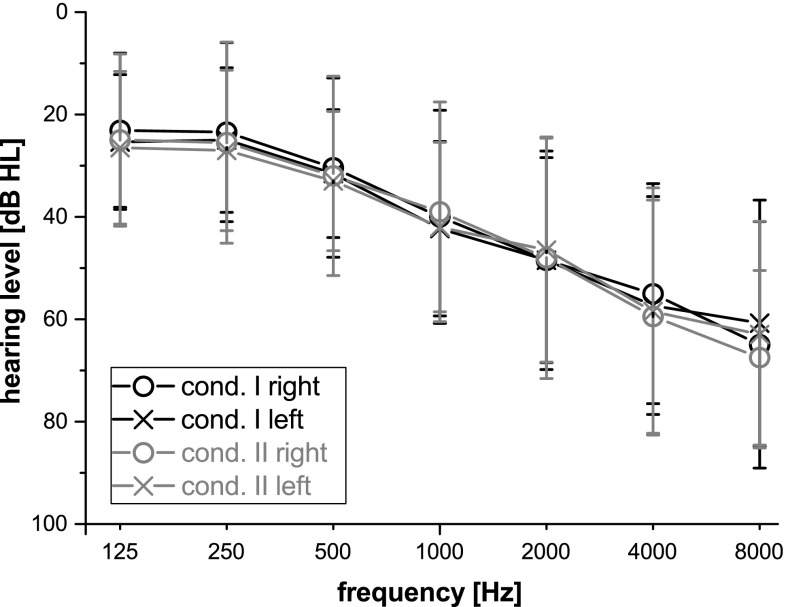
Mean pure tone audiograms and corresponding standard deviations of the included participants of both conditions of the study (condition I = *black color*, condition II = *gray color*)

At the end, 13 participants were included for condition I (mean age: M $=65.54$ years (SD $=8.24$ years), six female/seven male). One participant was excluded due to artifacts. For condition II, a total of 10 participants were included (mean age: M $=67.1$ years (SD $=7.92$ years), four female/six male). Here, one participant could not solve a part of the auditory task and the other one was excluded due to artifactual EEG data. Before the EEG session started the unaided hearing threshold was determined. For this, a standard audiometric examination using a clinical audiometer (tested pure tone frequencies: 0.25, 0.5, 1, 1.5, 2, 4, and 8 kHz) was conducted. The pure tones were presented monaurally via headphones. Figure 1 depicts the mean pure tone audiograms and the corresponding standard deviations of the included participants for both parts of the study.

### Hearing aid fitting

Commercially available behind-the-ear HAs connected to double ear-tips (double domes) were tested. The devices were fitted according to the hearing loss of the participant using a proprietary fitting formula. The HA amplification was set to an experienced level. The effects of the HA setting *directional speech enhancement* (DSE) on the participants listening effort were examined. The DSE setting is a combination of a directional microphone technique and a Wiener filter noise reduction.

Four HA settings were investigated to observe the differences regarding the listening effort. For this, the devices were fitted with the DSE feature set to a strong (DSEstr) and a medium setting (DSEmed). In a further setting the DSE feature was turned off (DSEoff), so that only the directional microphone setting was active. All settings were compared to an omnidirectional microphone setting (ODM) without additional noise reduction algorithms. Additionally, a short training session with each hearing aid setting was performed before the single tests started. This was done to guarantee that the participants understood and could solve the tasks.

### Stimulus materials and calibration of the auditory stimuli

To extract the possible listening effort correlates two conditions were generated. In condition I, the participants had to perform a task immediately after each stimulus presentation. The speech material was taken from a German sentence test [Oldenburg Sentence Test (OlSa); Wagener et al. (1999)], which is principally applied in clinical settings for the detection of the speech intelligibility threshold. Each sentence is spoken by a male voice and consists of the following structure: subject–verb–numeral–adjective–object (e.g., Peter buys three red cups). Additionally, there is no predictability of the context of the sentences (Wagener et al. 1999). The task is explained in detail in “Experimental design” section.

In condition II, the participants had to complete the task after the presentation of the speech material. In this part, the speech materials were two short stories taken from a German listening comprehension test [“Der Taubenfütterer und andere Geschichten”; Thoma (2007), level B1 (according to the Common European Framework of Reference for Languages: Learning, Teaching, Assessment; Modern Language Division (2007)] and also recorded by a male speaker. Each short story had a duration of approximately 10 min. Two HA features per short story were tested. For more details regarding the task see “Experimental design” section.

For both cases, the speech material was embedded in multitalker babble noise composed of international speech tokens naturally produced by six female voices (International Speech Test Signal (ISTS; Holube et al. 2010). Additionally, a cafeteria noise was added to the audio signals consisting of clattering dishes and cutlery (downloaded from a data base of auditory signals; Data Base: AudioMicro 2013). Furthermore, for condition II, the intensity of the cafeteria and the multitalker babble noise varied between two intensity levels in random time intervals between 5 and 15 s. The SNR was equally distributed over the conditions and the variations were the same for each participant.

The auditory stimuli were calibrated using a hand-held sound level meter (type 2250, Brüel & Kjær, Denmark) connected to a pre-polarized free field 1/2” microphone (type 4189, Brüel & Kjær, Denmark). To measure a single sound source (signal or noise), the loudspeaker for the calibration was placed 1 m in front of the sound level meter at the level of the participant’s head. Overlapping sound sources were measured at a distance of 1 m in the center of the loudspeakers. The levels for the OlSa and the short stories are stated for a single loudspeaker and the levels for the overlapping noises are given for all speakers.

To assess the fluctuating noise levels of the speech material, the “equivalent continuous sound level” ($L_{eq}$) was selected (Brüel and Kjær 2013). Furthermore, an A-weighting filter was applied as it is commonly used for the calibration of test stimuli for the sound field audiometry (BSA Education Committee 2008). The calibrated intensities were set to the following values: The intensities of the OlSa and the short stories were fixed at a conversational speech level of 65 dB $L_{Aeq}$ (Schmidt 2012). For the condition I, the ISTS noise had a level of 60 dB $L_{Aeq}$ and the cafeteria noise was set to 67 dB $L_{AFmax}$. To reveal a different listening environment, the ISTS noise used in condition II fluctuated between 64 and 66 dB $L_{Aeq}$. Likewise the cafeteria noise changed dynamically either at 64 and at 66 dB $L_{AFmax}$. These dynamic changes were used to reveal a realistic listening environment.

### Experimental design

To test the DSE feature, a total of four loudspeakers (Control One, JBL) were used. The speakers were positioned at a distance of 1 m from the participant’s head at $0^\circ$, $135^\circ$, $180^\circ$, and $225^\circ$ in the horizontal plane.

To generate different listening situations, two conditions were generated to extract the possible listening effort correlates.

*Condition I*

For this part, 50 OlSa sentences together with the ISTS noise were played at the frontal loudspeaker at $0^\circ$. For condition I, a total of 200 OlSa sentences were presented to test the four HA settings. Additionally, distracting noises were generated by two time-delayed ISTS and cafeteria noise sequences on each loudspeaker and played behind the participant at the positions $135^\circ$, $180^\circ$ and $225^\circ$. During the experiment, the task was to repeat words that were heard in the sentence played at $0^\circ$. A sinusoidal tone (1 kHz, duration: 40 ms) was added after each sentence to indicate the point of time where the participants’ response was expected, followed by a gap in the sentence stream with a duration of 5 s. The gap was only present in the sentence stream at the loudspeaker $0^\circ$. during the gap, the distracting noises were played continuously at $0^\circ$, $135^\circ$, $180^\circ$ and $225^\circ$. The responses were written down by the experimenter.

*Condition II*

In this part, the audiobook taken from the German listening comprehension test was played through the frontal loudspeaker $0^\circ$. The loudspeakers at the rear side (at the positions $135^\circ$, $180^\circ$ and $225^\circ$) presented simultaneously the two time-delayed ISTS noise sequences plus the cafeteria noise. The participant’s task was to answer simple questions related to the short story after the complete presentation of the audiobook, more precisely after presentation of all HA settings. This questionnaire consisted of 24 items. For each listening part, the participants answered between four and seven questions. Here, the participant was instructed to respond after the listening condition.

Condition I was designed to have a more controllable part. The participants had to repeat the sentence directly after its presentation. For this, it was easier to detect a drop in performance or to note if the participants quit the task. In condition II, the participants could listen to longer speech sequences, as it is usually the case in daily situations (e.g., listening to the radio or to a talk).

In both conditions, the four different HA configurations (a) DSEstr, (b) DSEmed, (c) DSEoff, (d) ODM were tested in a randomized order. Note also, that the presentation of condition I and II was randomized and the conditions were presented in separate blocks.

In both cases, the participants were asked to rate their perceived effort directly after each tested HA setting using a seven point scale (LE-Scale: no effort – very little effort – little effort – moderate effort – considerable effort – much effort – extreme effort adapted from Schulte (2009)) and their experienced speech intelligibility (SI-Scale: excellent – very good – good – satisfactory – sufficient – unsatisfactory – insufficient; Volberg et al. 2001). Additionally, the participants were asked to determine their preferred HA setting for a listening situation like the presented one after the completion of each part. During both conditions, the continuous EEG was recorded from the persons with hearing loss.

### Data acquisition and preprocessing

The EEG was recorded using a commercially available biosignal amplifier (g.tec USBamp, Guger Technologies Austria) with a sampling frequency of 512 Hz. Sixteen active electrodes were placed according to the international 10–20 system, with Cz as reference and a ground electrode placed at the upper forehead. The data were filtered offline using a linear phase finite impulse response bandpass filter from 0.5 to 40 Hz (filter order: 1000). For condition I of the study, a trigger signal indicated the onset and offset of each sentence. Thus, the EEG data could be analyzed during the presentation of the sentences (duration approx. 2 s, total of 50 sentences per hearing aid setting). After extraction of the EEG data for each sentence, artifactual EEG segments were rejected if the maximum amplitude threshold exceeded $\pm 70\,\upmu$V. The artifact free EEG-segments were recombined into a vector. This procedure was done for each EEG-channel independently. Finally, the recombined EEG-vectors were cut to an equal length of 80 s (minimum of 40 artifact free EEG segments in all EEG-channels × 2 s duration of a sentence). In condition II, artifacts were removed using a moving time window (duration: 2 s) and the same artifact threshold of $\pm 70\,\upmu$V. The artifact free EEG-segments were also recombined into a vector. The length of each EEG-vector was equalized to 320 s (minimum of 160 artifact free EEG segments in all EEG channels × window size of 2 s).

### Data analysis

The data analysis was performed using software for technical computing (Matlab2013a and Simulink, MathWorks Inc., USA). For the quantification of phase synchronization processes of the oscillatory EEG, the distribution of the instantaneous phase on the unit circle was investigated. The instantaneous phase $\phi _{a,b}$ of each artifact free recombined EEG channel was extracted by the application of the complex continuous wavelet transform. This means, the phase was extracted over the time samples of each EEG channel. Before the phase was extracted, the Hilbert transform was applied to the data to ensure an Hardy-spaced mapping.

Let

1 $$\begin{aligned} \psi _{a,b}(\cdot ) =|a|^{-1/2}\psi \left( \frac{\cdot -b}{a}\right) \end{aligned}$$

where $\psi \in L^2(\mathbb {R})$ is the wavelet with

2 $$\begin{aligned} 0< \int _{\mathbb {R}} |\Psi (\omega )|^2|\omega |^{-1} \mathrm{d}\omega < \infty \end{aligned}$$

$\Psi (\omega )$ is the Fourier transform of the wavelet, and $a,b\in \mathbb {R}$, $a\ne 0$.

The wavelet transform

3 $$\begin{aligned} \mathcal {W}_{\psi }:L^2(\mathbb {R})\longrightarrow L^2\left( \mathbb {R}^2,\frac{\mathrm {d}a\mathrm {d}b}{a^2}\right) \end{aligned}$$

of a signal $x \in L^2(\mathbb {R})$ with respect to the wavelet $\psi$ is given by the inner $L^2$–product

4 $$\begin{aligned} (\mathcal {W}_{\psi }x)(a,b)=\langle x,\psi _{a,b} \rangle _{L^2}. \end{aligned}$$

The instantaneous phase of a signal $x \in L^2(\mathbb {R})$ is given by the complex argument from the complex wavelet transform with the signal:

5 $$\begin{aligned} \phi _{a,b}=arg(\mathcal {W}_{\psi }x)(a,b). \end{aligned}$$

For the quantification of listening effort correlates, the mean resultant vector ${\bar{R}}$ was mapped to an exponential function (Fisher approximation of the Rayleigh equation). This mapping, was used as it is bounded between 0 and 1 and, compared to the previously examined angular entropy (Bernarding et al. 2012), it turned to be more robust against the later described sampling effect.

The mean resultant vector ${\bar{R}}$ of the phase values can be determined as follows. Assuming we have a set of unit vectors $x_{1}, \ldots , x_{N}$ with the corresponding phase angles $\phi _{n}, n = 1, \ldots , N$, then the mean resultant vector can be determined by

6 $$\begin{aligned} {\bar{R}}= \frac{1}{N}\left| \sum _{n=1}^N e^{\imath \phi _{n}}\right| . \end{aligned}$$

**Fig. 2:**
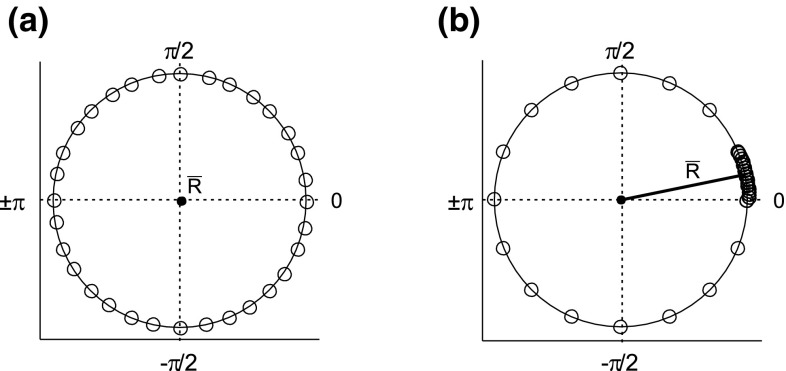
Schematic of the phase distribution of two theoretical data sets (*black circles*) together with their corresponding mean resultant vector ${\bar{R}}$ on the unit *circle* showing (**a**) a uniform distribution and (**b**) a non uniform distribution

The mean resultant vector ${\bar{R}}$ can be interpreted as a measure of concentration of a data set. The two schematics of Fig. 2 depict the phase values of a rather uniform (Fig. 2a) and a non uniform distribution (Fig. 2b) projected on the unit circle together with their corresponding mean resultant vector ${\bar{R}}$. If ${\bar{R}}$ is close to 0 (see Fig. 2a), then the phase values are more dispersed on the unit circle, which means that the data are distributed uniformly. Otherwise, if ${\bar{R}}$ is close to 1 (see Fig. 2b), then the phase is more clustered on the unit circle and has a common mean direction. Note that in large data sets the clustered phases are embedded in rather uniformly distributed phases, which is related to the sampling of the signal. If the data is sampled at consecutive and equidistant time points, we have a rather uniform distribution of the phases. If a phase reset occurs, then we have a clustering of the phases which is embedded in the preceding uniformly distributed phases. To be more robust against this sampling effect, the mean resultant vector is mapped to an exponential function.

The electroencephalographic correlate of listening effort can be defined for a specific scale *a* and a suitable auditory paradigm by

7 $$\begin{aligned} \text {objective \,listening\, effort\, (OLEosc)} \propto 1 - e^{- N {\bar{R}}^2}. \end{aligned}$$

A high value of the *OLEosc* corresponds to a higher listening effort.

To compensate for individual EEG differences, the individual’s *OLEosc* was normalized in the range [0,1] according to

8 $$\begin{aligned} OLEosc' = \frac{OLEosc-min(OLEosc)}{max(OLEosc)-min(OLEosc)}. \end{aligned}$$

### Statistical analysis

For a statistical comparison of the *OLEosc* with respect to the different HA configurations, a repeated measures analysis of variance (ANOVA) was applied to the data to detect differences on the listening effort measure regarding the applied HA settings. As post-hoc test a multiple pair wise comparison was performed with a Bonferroni adjustment. The Friedman Test was performed on the ordinal data of the LE- and the SI-scales as well as on the percentage of correctly repeated words. The post-hoc analysis of this data was performed using a multiple pair wise comparison with a Bonferroni adjustment.

## Results

The analysis was performed on the instantaneous phase extracted from the right mastoid electrode by the wavelet transform for a scale $a=40$, which corresponds to a pseudo frequency of 7.68 Hz (alpha–theta border). The scale $a=40$ and the electrode channel were identified in previous studies to reflect best correlates of an attentional effortful modulation. In these former studies, the listening effort correlates were gained from the evoked EEG activity (Strauss et al. 2010; Bernarding et al. 2010). There, it was shown that the best result can be obtained in the frequency range from 6 to 8 Hz. Additionally, in this lower frequency range were effects of an attentional, effortful modulation noticeable (cf. “Introduction” section).

For the analysis of the subjective listening effort scale, a number was assigned to each level of the LE-Scale (ranging from 1 = very little effort to 7 = extreme effort). Then, the mean and the standard deviation were calculated. The same was done to interpret the results of the subjective speech intelligibility scale. There the numbers were assigned to each level of the SI-Scale ranged from 1 = excellent to 7 = insufficient.

### Electroencephalographic and subjective listening effort estimation

A repeated measures ANOVA was conducted on the normalized *OLEosc* values to test if differences on the listening effort regarding the applied HA settings existed. There was a statistically significant effect of HA setting on the electroencephalographic estimate of listening effort for condition I [F(3,36) = 2.84, $p = 0.05$] and for condition II [F(3,27) = 4.57, $p=0.01$]. The results of the post-hoc multiple pair wise comparison with Bonferroni correction is shown in Table 1. Furthermore, significant differences regarding the *OLEosc* were found between the ODM setting and the DSEstr ($p=0.01$) as well as for the DSEoff ($p=0.04$) for condition I; and for condition II, the *OLEosc* was significantly different for the ODM and the DSEmed setting ($p=0.008$) as well as for the ODM and the DSEoff ($p=0.04$) setting.

**Table 1:** Results of the post-hoc multiple pair wise comparison (Bonferroni corrected), alpha level = 0.05

Hearing aid feature	Normalized *OLEosc*		LE rating		SI rating		Score	
	Condition I	Condition II	Condition I	Condition II	Condition I	Condition II	Condition I	Condition II
DSEoff × DSEstr	$p=1.00$	$p=1.00$	$p=0.74$	$p=1.00$	$p=0.96$	$p=1.00$	$p=1.00$	$p=1.00$
DSEoff × DSEmed	$p=1.00$	$p=1.00$	$p=1.00$	$p=1.00$	$p=0.69$	$p=1.00$	$p=1.00$	$p=1.00$
DSEoff × ODM	$p=0.04$	$p=0.04$	$p=0.017$	$p=0.01$	$p=0.017$	$p=0.011$	$p=0.009$	$p=0.246$
DSEstr × DSEmed	$p=1.00$	$p=1.00$	$p=1.00$	$p=0.83$	$p=1.00$	$p=1.00$	$p=1.00$	$p=1.00$
DSEstr × ODM	$p=0.01$	$p=1.00$	$p=3.6\times 10^{-5}$	$p=0.025$	$p=7.31\times 10^{-5}$	$p=0.0014$	$p=0.005$	*p* = 1.00
DSEmed × ODM	$p=0.07$	$p=0.008$	$p=0.0064$	$p=8.4\times 10^{-5}$	$p=3.22\times 10^{-5}$	$p=5.05\times 10^{-5}$	$p=0.0234$	$p=0.785$

There was also a statistically significant effect on the subjectively rated listening effort with respect to the tested HA setting for condition I, $\chi ^{2}(3)=22.04,\,p<0.001$, as well as for condition II, $\chi ^{2}(3)=20.14,\,p<0.001$. The multiple pair wise comparison showed significant differences with respect to the subjectively rated listening effort between the ODM and the other three HA settings (DSEoff, DSEmed, DSEstr) for condition I and condition II (cf. Table 1).

**Fig. 3:**
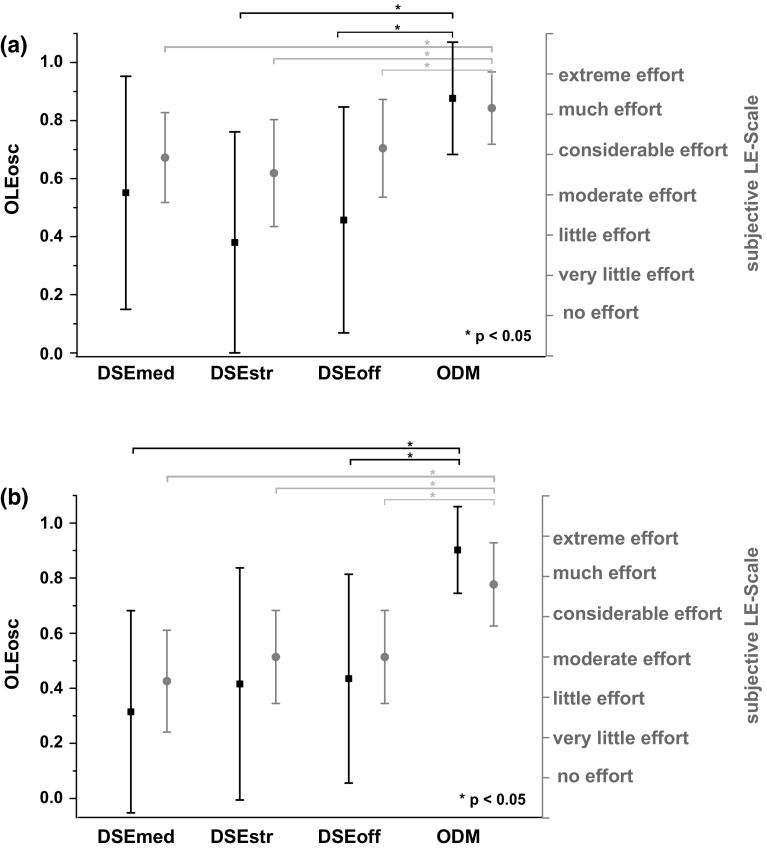
Mean and standard deviation values of the normalized electroencephalographic listening effort measure (*OLEosc*; *black squares*; *left* y-axis) and the subjective listening effort rating (*gray circles*; *right* y-axis) from the (**a**) condition I (mean over 13 participants) and (**b**) condition II (mean over ten participants). Note that higher values of the *OLEosc* indicate a higher listening effort

Figure 3 illustrates the mean results of the electroencephalographic listening effort measure (black squares; left y-axis) together with the mean results of the subjective listening effort rating (gray circles; right y-axis) over the four tested HA configurations for condition I (Fig. 3a) and the condition II (Fig. 3b) of the study. Note that higher values of the *OLEosc* indicate a higher listening effort.

Table 2 shows an overview of the preferred HA settings for condition I and II. It can be noted, that none of the participants preferred the ODM condition. Furthermore, in this preference data, no significant differences were noticeable (Friedman test). The electroencephalographic estimate of listening effort was highly correlated (Spearman’s correlation) with the subjectively perceived listening effort in all tested HA settings for condition I (r $=0.8$) and II (r $=0.94$). In the ODM setting, which should require the largest listening effort in this study, the participants had the largest listening effort with respect to the electroencephalographic estimate (*OLEosc*, condition I: M $=0.87$, SD $=1.93$; condition II: M $=0.90$, SD $=1.57$) and the subjectively rated listening effort (LE-Scale, condition I: M $=6.15$, SD $=0.90$; condition II: M $=5.80$, SD $=1.03$). The subjectively rated listening effort lies on the LE-Scale between considerable and extreme effort.

**Table 2:** Overview: number of preferred HA settings for condition I and II

	DSEmed	DSEoff	DSEstr	ODM	No preferences
Cond. I	4	4	3	–	2
Cond. II$^\mathrm{a}$	4.5	3.5	2	–	–

For these participants, each feature was scored with 0.5 instead of 1

$^\mathrm{a}$ In condition II, two participants preferred two HA features

### Speech intelligibility

**Fig. 4:**
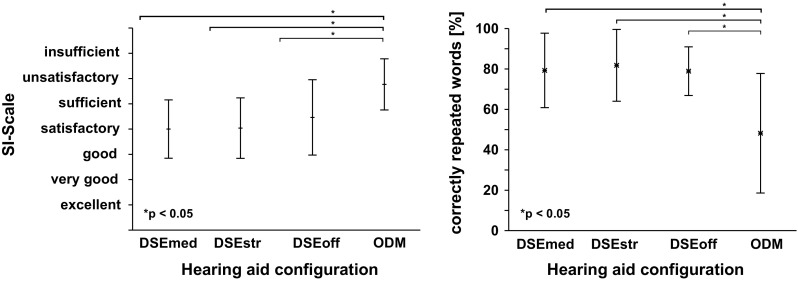
*Left* mean and standard deviation values of the subjective speech intelligibility scale for the condition I. *Right* mean and standard deviation values of the percentage of correctly repeated words for each HA setting for the condition I

The right side of Fig. 4 depicts the mean percentage of correctly repeated words over the four HA configurations of condition I of the study. Significant effects for the tested HA settings were found, $\chi ^{2}(3)=17.58, p<0.001$. Here, the multiple pair wise comparison was significant for testing the differences between the ODM and all other HA settings (DSEmed: $p=0.0234$, DSEstr: $p=0.005$, DSEoff: $p=0.009$). Besides the HA with the ODM setting, the participants reached a mean percentage of correctly repeated words around $80\%$ for the other three settings.

**Fig. 5:**
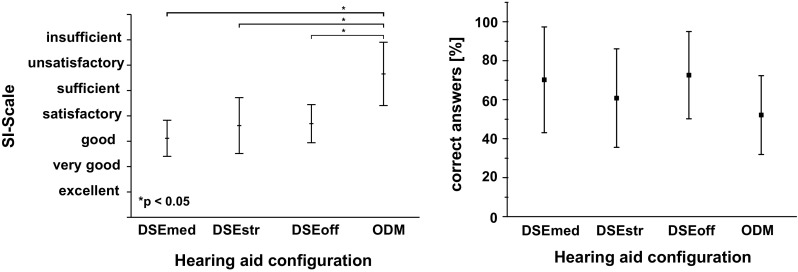
*Left* mean and standard deviation values of the subjective speech intelligibility scale for condition II. *Right* mean and standard deviation of correctly answered questions

The electroencephalographic estimate of listening effort and the word score data were also (negatively) correlated (Pearson’s correlation, condition I: r $=-0.96$). Regarding the SI-scales, there was a statistically significant effect with respect to the tested HA setting for condition I, $\chi ^{2}(3)=26.57, p<0.001$ and condition II, $\chi ^{2}(3)=22.88, p<0.001$. On the left side of Fig. 4 the mean results of the subjective speech intelligibility scale over the HA configurations for the condition I are shown. Again, the ODM achieved the poorest results. Significant differences between the SI-scales were found for the ODM setting versus DSEmed, DSEstr, DSEoff (DSEmed: $p=3.22 \times 10^{-5}$, DSEstr: $p=7.31\times 10^{-5}$, DSEoff: *p* = 0.017). The mean subjective speech intelligibility rating is between “sufficient” and “unsatisfactory” (SI-Scale, M $=5.77$, SD $=1.01$). In Fig. 5 (left), a similar behavior of the rated speech intelligibility can be seen for condition II. Again, only the difference between the ODM and the three other settings was significant (DSEmed: $p=5.05\times 10^{-5}$, DSEstr: $p=0.0014$, DSEoff: $p=0.011$). Compared to condition I, the speech intelligibility for the DSEmed, DSEstr and DSEoff configurations is slightly better rated, the SI is in a range between “good” and “satisfactory”. On the right side of Fig. 5, the mean and standard deviations of correctly answered questions is shown. Here, the differences between the four hearing aid settings were non significant.

### Effects of the presentation order on the electroencephalographic listening effort measure

**Fig. 6:**
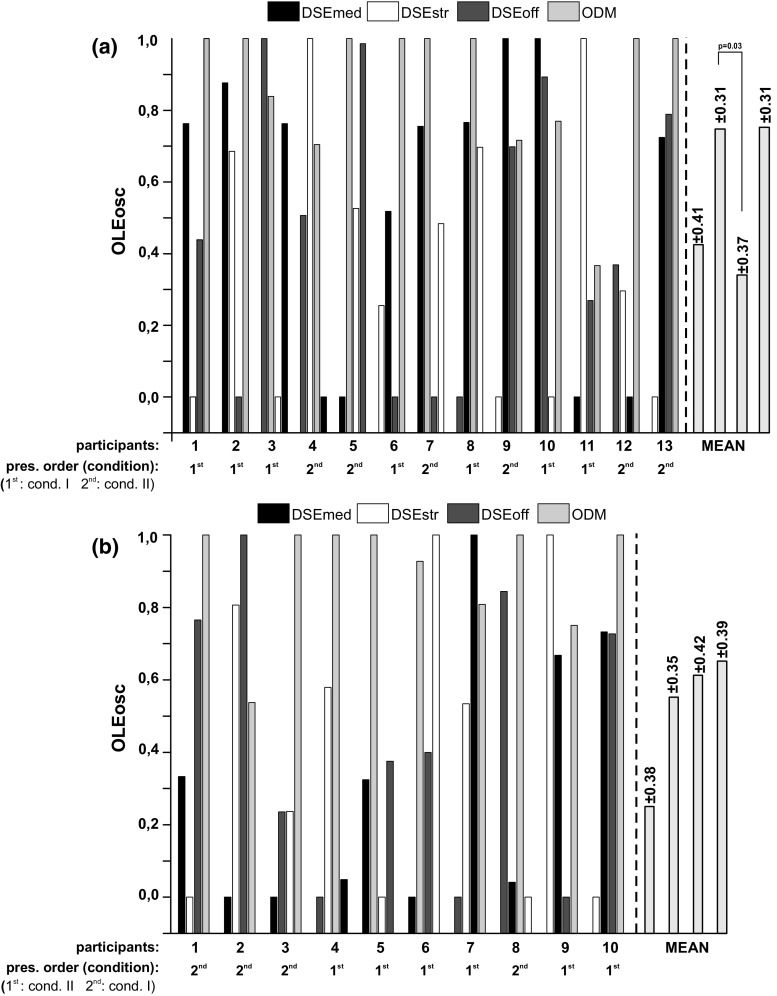
Individual and mean results of the normalized electroencephalographic listening effort measure sorted by the presentation order of the HA settings for (**a**) condition I and (**b**) condition II. Below the x-axis of each figure, it is also shown if the participants solved condition I or II in the first or second step of the experiment. Note that the ascent order tendencies for the participants 1 (condition I and II) and 10 (condition II) were related to the fact that the ODM condition, which was expected to require the largest listening effort, was presented at the end

To analyze possible influences of the measurement time on the *OLEosc*, like fatigue effects or a decrease of motivation, the *OLEosc* values for each participant were sorted according to the presentation order. After this, the mean and the standard deviation values were calculated for the two parts of the study. A repeated measures ANOVA was conducted on the *OLEosc* values to test if an effect of the presentation order on the listening effort measure exists. Only in condition I was a statistically significant effect noticeable [condition I: F(3,36) $=3.85, p=0.017$; condition II: F(3,27) $=1.76, p=0.17$]. There, the difference between the second and the third presentation was statistically significant ($p=0.03$). Note that this analysis was done additionally to the randomized testing of the HA settings during the experiments. The results of this analysis are depicted in Fig. 6.

The upper panel (Fig. 6a) represents the individual and the mean values of the normalized *OLEosc* sorted by the order of the applied HA configurations (x-axis, 1st to 4th setting, black to white bars) for condition I. The lower panel (Fig. 6b) shows the same, but for condition II. Besides participant 1 (condition I, Fig. 6a) and participant 10 (condition II, Fig. 6b), there is no increasing or decreasing tendency of the electroencephalographic listening effort measure related to the presentation order. In the case of the two aforementioned participants, the presented HA configurations required also an increased degree of listening effort (cf. Fig. 3, presentation order of participant 1: DSEmed, DSEstr, DSEoff, ODM; presentation order of participant 10: DSEstr, DSEmed, DSEoff, ODM). This means that the ODM setting was presented last and was expected to require the largest effort. The statistical analysis using presentation order as covariate showed similar results as the uncorrected ANOVA test (see Table 1): For condition I, the DSEoff versus ODM setting ($p=0.05$) and DSEstr versus ODM ($p=0.02$) were significantly different; as well as for condition II, the DSEmed versus ODM setting ($p=0.008$). Here, the DSEoff versus ODM setting had a significance level of $p=0.06$.

## Discussion

The main objectives of this study were: (1) to estimate listening effort by means of EEG data; and (2) to investigate the effects of different HA configurations on the listening effort.

The most important finding of this study is that the new electroencephalographic estimate of listening effort reflects the subjectively perceived effort of the participants with hearing loss in both listening conditions.

The results indicate that a higher value of the proposed listening effort measure *OLEosc*, mirrors a higher subjectively rated effort. This suggests that the distribution of the instantaneous phase of the EEG in the range of the theta band is correlated with cognitive effort, which means that the phase is more clustered for a demanding condition. Regarding neuronal entrainment, the cortical oscillations can be modulated by an exogenous stimuli or an endogenous source (Weisz and Obleser 2014).

Peelle et al. (2013) showed in an MEG study using noise vocoded speech that slow cortical oscillations become entrained when linguistic information is available. They argued that this phase-locking relies not only on sensory characteristics, but also on the integration of multiple sources of knowledge, like top-down processes. Similar to these findings, Kerlin et al. (2010) found in their EEG study an attentional enhancement of the 4–8 Hz signal in the auditory cortex. They discussed that for a successful encoding of the speech, the phase-locked cortical representation of the relevant speech stream is enhanced via an attentional gain mechanism. Regarding these aspects, it can be interpreted that the EEG phase clustering in the frequency range of the theta band reflected in a high *OLEosc* value is due to an increased effortful endogenous modulation.

Furthermore, we can hypothesize that the defined measure can be linked to our previous findings of the phase synchronization stability of evoked responses (ERPs) via the phase reset theory (Strauss et al. 2010; Low and Strauss 2009; Corona-Strauss and Strauss 2017). In Low and Strauss (2009) the connection between the ERPs and the EEG was investigated. There, tone-evoked ERPs were recorded from participants focusing their attention on a specific target as well as a recording of an unfocused condition. It was shown that an artificial phase reset at a specific frequency in the range of the alpha-theta band of the unfocused data resulted in an increased N1 amplitude. These modified N1 amplitude was similar to the one gained from the attentional condition. Additionally, it was demonstrated that smaller variations in the instantaneous phase of the EEG lead to an enhancement of the attention dependent N1 amplitude (cf. “Introduction” section). Regarding this ERP phase clustering due to focused attention, we can hypothesize that there is a similar attention related modulation of the ongoing EEG. We assume that both processes originate from the same attention networks (Raz and Buhle 2006).

The results show, that besides the correlation between the *OLEosc* and the subjective listening effort rating scale, also a correlation between the *OLEosc* and the speech intelligibility score exists. Furthermore, a benefit of the directional microphones (with and without noise reduction algorithm) over omnidirectional microphones was illustrated. Ricketts (2005) discussed in a review that the use of the directional microphone technique can be an advantage for particular listening environments, for instance, environments where an increase of the SNR between 4 and 6 dB leads to an adequate level of speech intelligibility. Related to the fact that directional microphones effectively improve the SNR, the audibility of the speech signal is enhanced which is accompanied by a reduced listening effort. On the other hand, Hornsby (2013) found no additional benefit of the usage of a directional processing mode. There, the listening effort was assessed by subjective listening effort ratings, word recall and the visual reaction time gained from a dual-task paradigm. The next step would be to investigate the *OLEosc* and the subjective listening effort rating at an individually adjusted speech level or at an SNR where the speech is in all the test modes highly intelligible. In such cases, the listening effort required to achieve a similar speech level could be examined (Brons et al. 2013). In addition, significant differences between the three directional microphone settings, namely an improvement of the noise reduction algorithm, could not be shown. Neither by the subjective rating scales and the speech scores nor by the *OLEosc*.

Sarampalis et al. (2009) examined a benefit of a noise reduction algorithm on the listening effort. They tested people with normal hearing sensitivity with processed and unprocessed speech samples. However, in this study, solely the noise reduction setting was tested and not a combination of a directional microphone and a noise reduction algorithm. Regarding this aspect, it could be possible that in the current study the additional effects of the noise reduction algorithm on the listening effort are not trackable with the applied experimental paradigm. Additionally, the results of the individually preferred HA settings, showed no clear trend of an overall favored HA setting. This could be related to individual preferences, like a highly individualized noise annoyance (Brons et al. 2013). It is also possible, that the differences between the HA settings are marginal and therefore not detectable with the applied paradigm. Thus, a general recommendation which of the tested noise reduction settings reduces the listening effort maximally cannot be made.

Although a randomized presentation order of the HA settings was applied, we can not fully exclude possible order effects on the subjective as well as objective estimates as the randomization was not fully balanced. However, the (individual) results show no systematic change over the measurement time, like an increasing or a decreasing tendency of the *OLEosc* measure. Such tendencies could be expected due to fatigue effects (Boksem et al. 2005), stress or a lack of concentration according to the measurement time. As a result the participants would either spend an additional effort to solve the auditory task or they lose the motivation to perform the task (Sarter et al. 2006).

Comparing the perceived speech intelligibility and listening effort of condition I and II with each other, it can be noted that there is a tendency of increased values for condition I. This means, condition I required slightly more effort and also the audibility was reduced in this case. Nevertheless, the the difference between condition I and II for the same participants (ten participants) was not significantly different. At a first glance, this result is not expected as a better SNR was used in condition I. This means, related to the physical part of the speech discrimination process, the speech intelligbility should be poorer for condition II. However, if speech information is inaudible, the cognitive system makes also use of context and linguistic information to support the speech understanding, i.e., the context information can help to interpret the missing auditory information (Edwards 2007). In condition I, sentences from a speech intelligibility test were used, which had no predictability of the context of the sentences (duration approx. 2 s). In the second condition, the speech material consisted of a continuous audiobook. There, the participant listened 5 min to each part of the audiobook. We could interpret, that in the second case, the participant could make use of the context information to support the speech understanding. Furthermore, the responses were expected after listening to the whole part of the audiobook and not directly after each sentence. Thus, we could assume, that they realized how much of the information was inaudible for them. In the other condition, the listening period was much longer and the participants had to answer text related questions. With respect to this aspect, we could assume, that the participants had a more vague idea of how much of the information they really missed.

An advantage of the new measure is that we obtain the listening effort directly during the auditory task. The benefit of such an objective method is, that it is not subjectively biased. Additionally, the listening effort could be measured continuously on finer levels compared to a discrete rating scale with a limited number of categories. However, the investigation if the *OLEosc* can differentiate marginal effort differences was beyond the scope of this study.

Nevertheless, we still have to test this measure in different HA configurations and it has also to be validated in future studies, which are more related to the standard clinical practice on an individual basis. Further work should also analyze the temporal progress of this measure during the listening process.

## Conclusion

We have presented in this study a novel electroencephalographic method to estimate listening effort using ongoing EEG data. The results suggest that the new listening effort measure, which is based on the distribution of the instantaneous phase of the EEG, reflects the exerted listening effort of people with hearing loss. Furthermore, different directional processing modes of the HAs with respect to a reduction of the listening effort were tested. The new estimate of listening effort indicates that a directional processing mode can reduce the listening effort in specific listening situations.
